# Development of Water Culture Tourism of Mountain Ethnic Culture Based on 3D Image Technology

**DOI:** 10.1155/2022/5465488

**Published:** 2022-06-23

**Authors:** Chuangle Guo, Xinlu Shi

**Affiliations:** Chengdu University of Information Technology, Chengdu, Sichuan 610000, China

## Abstract

The study of human settlement environment, especially the complete study of human settlement environment in mountainous region, is a huge systematic project, which involves almost all aspects of human knowledge system at the semantic level. It is an important task to establish a systematic cognitive model of human settlement environment and carry out practical verification. In recent years, due to the rise of tourism real estate, mountain tourism real estate, as a type of tourism real estate, has attracted people's attention, and its hidden economic, social, and ecological benefits make it develop rapidly. The purpose of this study is to improve the theoretical system of tourism real estate landscape planning and mountain landscape resource protection system with 3D image technology, which has important theoretical and practical significance for exploring the sustainable development method of mountain tourism real estate landscape. This study locates the research type of mountain tourism real estate, and it is the first time to study mountain tourism, explores the specific planning of mountain tourism real estate development measures, and preliminary establish the theoretical system of mountain tourism real estate development. It brings a new idea for the development of mountain tourism real estate and puts forward the direction and method of planning.

## 1. Introduction

With the development of the economy and the improvement of people's living standards , many buyers are simultaneously turning to the tourism industry, which has rapidly spread from large- and medium-sized cities to small-sized cities. Therefore, vacation has become the main form of tourism in a short time [1]. In order to meet the demands of consumers, real estate has seized the opportunity. A large number of resorts and hotels have been established in scenic resorts along the coast [2]. However, too fast development tends to lead to problems such as imperfect infrastructure, poor conceptual overall planning, and destructive landscape ecological environment. An unsustainable economic growth model and consumption model will inevitably bring severe challenges and threats to human ecological environment and social development. Especially for the tourism industry, the impact is very far-reaching [3, 4].

 Mountainous area is a terrain with uneven surface distribution and elevation difference reaching a certain threshold. However, from the perspective of human social attributes, mountain characteristics are actually an important cause of spatial differentiation [5]. [6, 7]. . Of course, this reason is not direct. No matter from the perspective of a spatial scale, the spatial differentiation of social people is generally manifested as concentration or dispersion on a large spatial scale, thus deriving the urban living environment. People create living environment, and living environment affects people's behavior. This feedback is particularly strong in mountain man environments [6,7]. From the present single perspective, the ultimate problem of human settlement is to further study the relationship between spatial heterogeneity and human settlement construction, so as to realize the harmonious coexistence and sustainable development of mountain environment and human system. The settlement environment here is not a city alone, but an organic community composed of regional cities, towns, communities, and even buildings [8, 9].

With the continuous development of tourism real estate, how to carry out the construction of landscape environment of tourism real estate, to achieve the harmonious balance and sustainable development of economy and social culture by landscape ecology, is a problem that we are facing at present and need to solve. Tourism real estate development is a collection of landscape environment and the combination of tourism real estate planning cross domain [10]. For now, in regard to the development of tourism real estate in our country, we mainly consider three aspects. One aspect is the economic interest. The developer is the main purpose of the development in order to obtain the maximum economic return. This is cannot change. Another aspect is focused on tourism real estate planning study, mainly producing industry planning. The third aspect is the landscape planning research of tourism real estate itself, mainly studying the landscape ecological planning of tourism real estate. However, considering these aspects alone, we cannot carry out effective and reasonable development and construction [11]. We must stand on the basis of the overall landscape environmental benefits and take ecology, landscape science, environmental landscape ecological sustainable development of tourism geography culture, and other related theories, an overall evaluation of the tourism real estate development [12]. The purpose of this study is to discuss how to carry out the tourism real estate landscape development approach according to the environmental conditions of specific regions through relevant theories [13].

New theories promote the development and evolution of new technologies, and new technologies often, in turn, promote the development and innovation of theories. The main technology platform of this study is based on 3D image processing technology, which is a beneficial attempt to innovate the discipline methodology of architectural planning. This study tries to find a new scientific research model of human settlement environment and supports the scientific and expansibility of human settlement environment with new methods and solid scientific logic. As far as the current situation is concerned, 3D image technology has been applied in the field of urban planning, but this kind of research lacks an integrated, systematic, and theoretical research perspective, especially the research on the cultural tourism of famous ethnic groups in mountain areas, which makes it possible for this research to achieve some original results. The research significance of this study is summarized as follows:

1. With the development of tourism real estate, the infrastructure of tourism resort is constantly built and improved, and a large number of construction are poured into coastal, lake, mountain, and other landscape tourism areas, resulting in the destruction of many original landscape environment. At the same time, due to the over-development of tourism real estate in the local area, the local traditional social culture gradually declined or even disappeared. Therefore, to maintain continuation and development of landscape culture resources, we must first consider the future development of tourism real estate is the problem; only through reasonable and sustainable development of protection principles can we make our eternal vitality and unique tourism real estate landscape.
2. In the 1990 s, China began to tourism real estate in 1992. The State Council approved the construction of 12 state-level tourist resorts, and so far, various types of tourist real estate have developed rapidly across the country. However, compared with developed countries such as Europe and the United States , tourism real estate in China started late; the related theoretical system is relatively backward; in order to establish perfect theory system, we must combine landscape science, environmental, ecology, and other related theory, to explore suitable for China's national conditions of tourism real estate development real principles and methods, so as to promote the perfection of the theory on tourism real estate development in China [16].
3. The development of tourism real estate will certainly promote the development of local economy, which is of course beyond doubt. However, we also want to scientifically use of reasonable planning and improve the utilization rate of resources, to maximize the local resource advantage to tourism as today's popular industry, not only brought the rapid development of economy but also benefits the local people, and a more solid foundation has been laid for the construction of socialist avenue [17, 18].

## 2. Related Work

Tourist real estate in the south of France and Naples, Italy, had its earliest forms before the nineteenth century. In 1839, Lord Brougham, the British Prime Minister, built his own villa on the Bay in southern France and praised it, thus arousing the great interest of most British people in the bay. In 1858, the French poet, Conneau, sent a letter to the wife of Napoleon III and once again made the southern coast of France a holiday hot. Due to the establishment of villas and hotels and various entertainment facilities, the most famous leisure and health resorts are made here in Europe, thus attracting many nobles to gather here. After World War I (1918), before the advent of the Great Depression, the traditional Puritan morality collapsed and the hedonism of the jazz age became popular. The value of this land on the south coast of France was once again favored by Americans, and American jazz culture replaced the original aristocratic coastal vacation culture [19].

In the early 1980s, the concept of timeshare vacation exchange began to spread all over the world, mainly developed capitalist countries in Europe. During this period, a large number of investors and developers in this area, due to the increasing product, make product business model more clear, relevant law also constantly improves and forms a complete set of services, and management industry and exchange industry also developed rapidly mature; according to relevant data, in 1999, the global timeshare property sales was as high as 6.72 billion dollars, and the United States accounted for 55%, among which the sale of rooms accounted for 50.4%. The world's largest resort, Condominium International (RCI), is an American company founded in 1974. Headquartered in Indiana, USA, the company is the first in the world to introduce the concept of timeshare exchange. As one of the global vacation rental market leader, RCI has more than 3 million members worldwide and covers 73,000 resorts worldwide [20, 21]. In the past three years, RCI vacation membership has also increased by 25 percent. In recent years, Japan, South Korea, India, Thailand, Philippines, Singapore, Malaysia, and other Asian countries have also played a tourism holiday brand. Resort hotels, leisure, health, leisure, hot spring clubs, weekends, holidays, etc., are also the heart of the development of the world economy. International financial investors and hotel management and investment institutions have also been involved, making the forward development of tourism real estate more hopeful [22].

China's tourism real estate started late, starting with timesharing resort hotels in Hainan in the 1990s. With the development of China's reform and opening up, Hainan has its unique tropical never winter island, beautiful scenery, pleasant climate, and fresh air, which attracts a large number of real estate developers and presents the real estate demand in Hainan. During this period, Hainan's tourism real estate accounted for 40% of the national market share. Hainan's real estate development mode integrating leisure, and vacation tourism is overturning the traditional real estate development mode. Hainan has always been regarded as a paradise for tourism and vacation. Tourism real estate started earlier [23]. However, after the real estate bubble in 1992 and 1993, tourism real estate was really taken seriously vacant commercial housing [20]. Starting in 1999, Hainan has spent seven years of processing after the bubble burst Commercial housing backlog. In the whole process, tourism real estate has played a big role This road is proved that the tourism real estate development model not only can make the Hainan real estate out of the shadows of the bubble but also make it step-by-step prosperity. In 2005, Hainan closely combined real estate and tourism with tourism real estate as the main brand [24], creating a peak of tourism real estate development in Hainan. Hainan has always been regarded as a paradise for tourism and vacation, and tourism real estate started earlier. However, after the real estate bubble in 1992 and 1993, tourism real estate was really taken seriously. The bubble economy has been the pain of Hainan real estate for a long time, which directly brought about a large number of half-completed projects and idle commercial houses. In the whole process, tourism real estate has played a significant role. This road proves that the development mode of tourism real estate can not only make Hainan real estate out of the shadow of bubble but also make it gradually to prosper. In 2005, Hainan closely combined real estate and tourism, with tourism real estate as the main brand, creating a peak of tourism real estate development in Hainan [25, 26].

**Figure 1:**
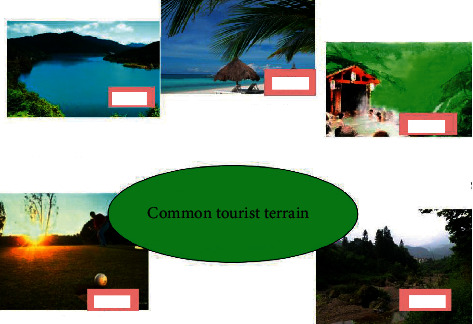
The common tourist terrain.

Figure 1), [27]. . According to the characteristics of the surface, the surface can be divided into four basic forms: plain, hill, plateau, and mountain (as shown in Figure 1)1. The surface has a large undulating area, mostly in the area of tectonic movement and external force activity, and the geological structure is complex, which is characterized by large absolute height and relative height [27]. Mountain has broad sense and narrow sense, general mountain, highland mountain, and hill three parts, among which narrow sense mountain only refers to very low mountain. Since the measurement process of spatial uniformity itself is a continuous cycle, the research object of this study is the generalized mountain tourism [28].

On the contrary, while the theory and practice of human settlement environment science are flourishing, as a cross-sectional methodology, the related theory and empirical research of 3D image technology is also flourishing in the geoscience, although the research of 3D image mountain tourism is also in a stage of exploration and research. It can be noted that, so far, the relevant research on mountain tourism is mainly focused on the natural field (landform, water system, natural landscape, etc.). From the perspective of semantic model, the object and category of mountain cultural tourism research are quite different from that of human settlement environment research, which can only be used for reference in methodology [29]. Therefore, the research of human settlements info-tupu is inherently exploratory and has certain risks. However, it is precisely because of the interdisciplinary nature of the academic field that it is easiest to achieve fruitful results between scientific fields. It is worth noting that there are still some areas for improvement in the current research:

1. Parallel: the first is environment science and the information mapping theory of parallel; so far, the living environment of scientific research achievements of theory and practice has widely appeared, geological information of map-related research also gradually prospers [30].
2. Object discretization: at present, the outstanding problem of human settlements environment-related research is object discretization, and there is lack of effective integration. The research methods are flat and descriptive, and there is no effective methodology and technical model for the study of human environment system. The object discretization is mainly manifested by the fuzzy definition of the object of human environment system or the semantic repetition and logical crossover in the definition of research objects.
3. Mountain plains: the main problem is the lack of summary of the characteristics and rules of living environment; just the plain human settlement environment model and the design method to are used to study mountain problems [31]. The grid layout of mountain cities has become popular in recent years. Therefore, this study proposes the research on water culture of mountain ethnic culture tourism development based on 3D image technology [31].

The contribution of this study is as follows:

1. Improve the theoretical system of tourism real estate landscape planning and mountain landscape resource protection system with 3D image technology
2. This work has important theoretical and practical significance for exploring the sustainable development method of mountain tourism real estate landscape

## 3. 3D Image Technology-Based Water Culture of Mountain Ethnic Culture Tourism

### 3.1. Water Culture of Mountain Ethnic Culture Tourism

The research is a typical cross-cutting research, which has the following key issues to be solved at the theoretical and technical level: (1) semantic (conceptual) composition and logical relationship sorting (object) of (mountain) human settlement environment system; (2) the constitution and internal contradiction of human settlement system (structure); (3) identification and expression model of human settlement environment system (mountain) (technology); (4) index system and numerical modeling (method) of (mountain) human settlement environment; (5) (mountain) regional verification of spatial information Atlas of mountain human settlement environment (test); (6) law condensing and model innovation (summary). To analyze and express the living environment through the spectrum of spatial information graph of the living environment, its key logical order is as follows: clear the research object, analyze the object structure, establish the expression model, establish the analysis model, establish the empirical model, and propose the planning model.

Among the six key links, the most important problem is the living environment of semantic model because when the former human settlement environment still stays on the concept description, it is an open system; combined with the related discipline, each holds one word and is not easy to use unified agreed to the key words to describe key logic structure of living environment. As a result, the solutions to these key problems are largely subjective. In the practical process, it is necessary to combine theory with practice, in order to carry out comprehensive research and analysis on the basis of the existing theoretical and practical achievements. At the technical level, the study takes 3D image technology as the platform, comprehensively integrates various research methods, and forms a research framework of mountain culture tourism.

### 3.2. 3D Image Technology

Convolutional neural network is one of the important branches of deep learning. The super-resolution technology based on convolutional neural network mainly relies on the rich operations of neural network such as convolution, deconvolution, pooling, and activation. Convolutional layer is the basis of neural network. Through the convolutional kernel (convolutional template), input signals can be extracted from different levels, depths, and even different frequency bands of information. The convolutional kernel is similar to various operator templates in traditional algorithms, such as gradient operator and smoothing operator. However, traditional template operator values are fixed, and specific features can only be extracted according to specific settings. Different from this, convolutional templates in neural networks only need to be initialized and then automatically updated through the iterative optimization of the network. At present, the convolutional kernels are divided into 2D convolution and 3D convolution, and their sizes are generally odd.

In the network design architecture, in addition to the linear or nonlinear processing of data, there are many specialized normalized layer operations that are helpful for network training convergence. For example, the layer normalizes data in channel direction, and the group norm divides channels. The most effective method is Batch Norm (BN), which refers to subtracting the mean and dividing by the variance in the ordinary sense, while BN is to normalize the data of eachbatch. In addition to subtracting the mean and dividing the variance, scale transformation parameter *γ* and offset parameter are also introduced. This can not only make the output data of the upper layer conform to normal distribution but also keep the characteristic structure of the output of the upper layer unchanged. The following is the derivation of the specific formula:

(1) $$\begin{array}{c} u=\frac{1}{N}\sum_{i=1}^{N}x_{i}, \end{array}$$

(2) $$\begin{array}{c} \sigma^{2}=\frac{1}{N}\sum_{i=1}^{N}{\left(x_{i}-u\right)}^{2}, \end{array}$$

(3) $$\begin{array}{c} {\hat{x}}_{i}=\frac{x_{i}-u}{\sqrt{\sigma^{2}+\epsilon }}, \end{array}$$

(4) $$\begin{array}{c} y_{i}=\gamma {\hat{x}}_{i}+\beta . \end{array}$$

The above super-resolution method is to build some basic layers through the network, based on these basic operational layers. At present, convolutional neural networks, used to achieve image super-resolution, can be roughly divided into series and parallel networks, residual network, pyramid network, recursive network, dense connection network, and generative adversarial network and the combination between them. The structure of series and parallel networks is relatively simple and the input of low resolution rate graph. For example, the serial convolutional structure of the network should not be too deep through the direct output of stacked convolutional layers. It is an efficient structure and has significant advantages over other structures, whether used for classification tasks such as recognition or regression tasks such as super-resolution reconstruction. The selection of network training optimizer mainly includes batch gradient descent (BGD), stochastic gradient descent (SGD), minibatch gradient descent (MBGD), and momentum optimization algorithm. The momentum learning method weighs the importance of the previous gradient and the current gradient on the basis of SGD, and the formula is expressed as

(5) $$\begin{array}{c} W_{t}=W_{t-1}+\alpha V_{t}, \end{array}$$

(6) $$\begin{array}{c} V_{t}=\beta V_{t-1}+\left(1-\beta \right)dW\;. \end{array}$$

The RMSprop optimizer can adaptively adjust the learning rate by using the gradient accumulation of previous training, and its updating formula is

(7) $$\begin{array}{c} W_{t}=W_{t-1}+\frac{\alpha }{\sqrt{\theta_{i}+\epsilon }}dW\;, \end{array}$$

(8) $$\begin{array}{c} \theta_{i}=\beta \theta_{i-1}+\left(1-\beta \right){\left(dW\right)}^{2}. \end{array}$$

Adam optimizer combines a compromise version of momentum optimization and RMSprop optimization, which is the most stable among all optimizers at present. Its optimization strategy can be summarized as

(9) $$\begin{array}{c} g=\frac{1}{m}\sum_{i=1}^{m}\frac{\partial L\left(y^{i},f\left(x^{i},w\right)\right)}{\partial w}, \end{array}$$

(10) $$\begin{array}{c} v_{t}=\beta_{1}v_{t-1}+\left(1-\beta_{1}\right)g, \end{array}$$

(11) $$\begin{array}{c} r=\beta_{2}r+\left(1-\beta_{2}\right)g^{2}, \end{array}$$

(12) $$\begin{array}{c} W_{t}=W_{t-1}+\frac{\alpha }{\sqrt{r+\epsilon }}v_{t}. \end{array}$$

**Figure 2:**
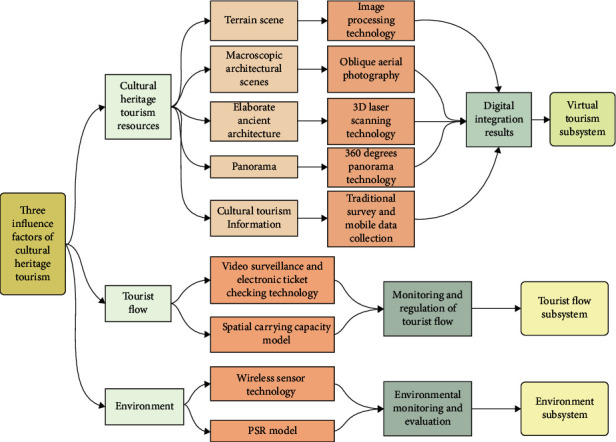
3D image technology-based water culture of mountain ethnic culture tourism framework.

Based on equations (1)–(12), Figure 2 gives 3D image technology-based water culture of mountain ethnic culture tourism proposed in this study.

## 4. Experimental Results and Analysis

### 4.1. Introduction to Experimental Dataset

This study mainly adopts the method of corpus research and combines quantitative statistics with qualitative analysis. First of all, we collected news texts from People's Daily for consecutive months and established a large database. Then, according to the research purposes of different chapters, we extracted samples from the main corpus and built a secondary corpus. For some chapters, we also classified and collected them according to the needs. Then, we sorted all the subcorpora in each chapter of the newspaper into text documents to establish a pair of subcorpora.

### 4.2. Experimental Results' Analysis

**Figure 3:**
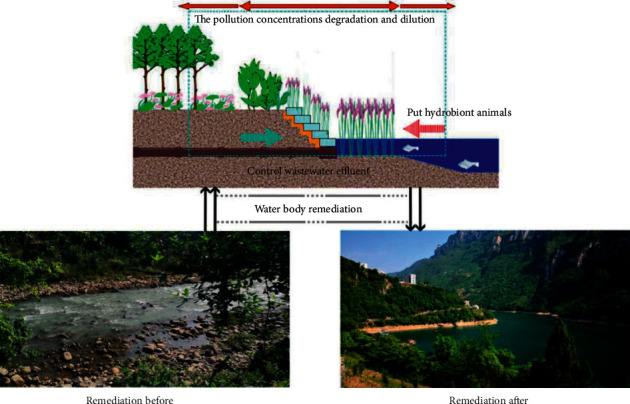
Visualization results of water restoration in mountain tourism area.

There are many kinds of tourism resources, including viewing and recreation resources, ecological environment resources tourism services, facilities and service level, hotel catering, leisure entertainment, etc. All kinds of tourism resources are leisure tourism areas developed by vacationers or tourists and are the material basics for entertainment, sports, fitness, and other activities. The type, quality, and scale of tourism resources directly determine the attraction and development potential of the resort. Through the transformation of buildings, vegetation, and water bodies in Shinan Water culture tourism zone, the efficiency of water-bearing forest is improved. The dust-holding capacity of rivers is conducive to the degradation and dilution of concentration of some pollution sources and the improvement of water ecosystem function and water environment quality. The comparison before and after the transformation is shown in Figure 3.

**Figure 4:**
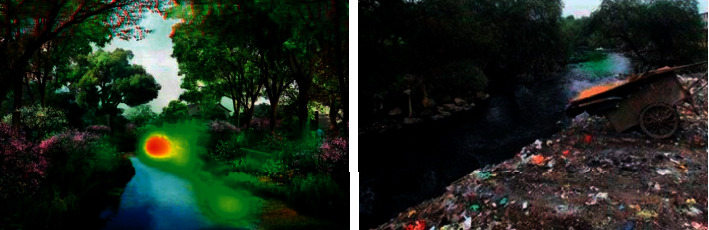
Focus on hotspot distribution before (b) and after (a) mountain water resource rehabilitation design project.

The size of the derived agents (artificial and social) of the corresponding human settlements also increases. In a long period of history, the suitability state of mountain tourism area is approximately unchanged, that is, during this period, the adaptability of human settlement environment is mainly passive adaptation. If the object of human settlement is simplified as topographic and geomorphic environment, the suitability of the object of human settlement can be approximately regarded as a fixed value. With the development of human society, especially after the arrival of industrialization process (usually also manifested as rapid urbanization process), the characteristics of the subject and object of human settlement environment have undergone tremendous changes. The focus on hotspot distribution before (right) and after (left) mountain water resource rehabilitation design project is given in Figure 4.

**Figure 5:**
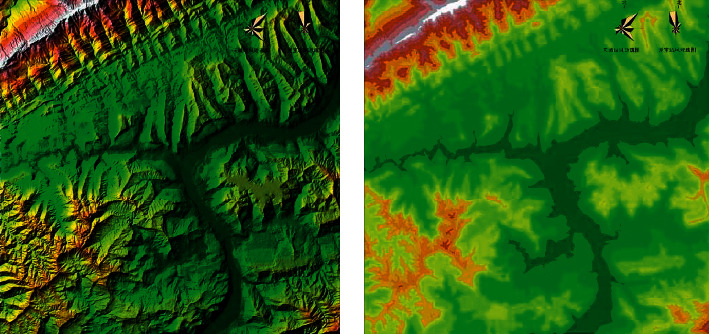
Landform space form (a) and Spatial form of elevation distribution (b).

From Figure 5, we know the topographic and geomorphic characteristics of the planning area (the attribute domain is uncertain). The planning area is located in the deep hill and low mountain area, with broken topography, large terrain slope, lack of large flat land, poor urban land conditions, and low land utilization rate. The coefficient of buildable land in the planning land is about 40–50%, and the elevation distribution characteristics is of the planning area (attribute field is 1251). The altitude span of the planning area is large, ranging from 115 to 1366 meters at the lowest level. Combined with the topography and geomorphology, it presents an obvious platform elevation (M) ranging from 115–215, 215–415, 415–615, 615–915, and 915. The proportion above was 11%, 39.1%, 33.4%, 13.4%, and 13%, respectively.

**Figure 6:**
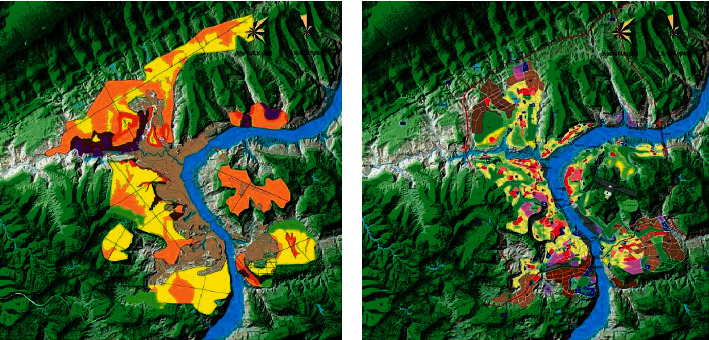
Space form of engineering geological conditions (a) and spatial form of land use planning (b).

In addition, from Figure 6, we know the characteristics of engineering geological conditions in the planning area (attribute field is 3). There are 18 dangerous rocks and 15 landslides in the planning area which have passed the risk assessment of geological disasters. The planning space is divided into three types: forbidden area, cautious area, and suitable area. Forbidden area is mainly distributed in the upper flat on the north bank of Zhuxi River and the area at the foot of Lion Mountain, and careful area mainly distributed in the north of ShenMingBa, including the building of the area, is not prone to geological disasters and low rock. The structure of urban land is a multicenter group layout structure, consisting of 8 groups and 3 areas (Longbao Tiancheng Five Bridges). The total planned land area is 80.99 km2, and residential land accounts for 25.1% in terms of attribute composition. Public facilities, industrial storage, external transportation, roads, squares, municipal public facilities, green land, and special land accounted for 14.7%, 21.4%, 2.8%, 9.8%, 13.3%, 5.6%, 13.8%, and 2.3%, respectively.

**Figure 7:**
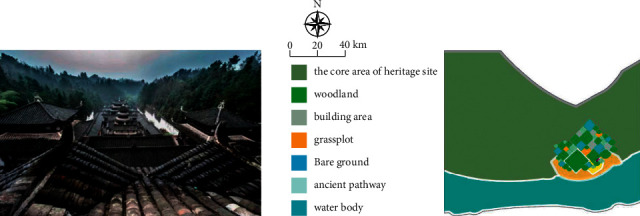
Cultural landscape pattern design project after renovation.

Due to the unique tourism resources and huge development potential of tourist resorts, it is of great significance to study and explore the development of famous cultural tourism in mountain areas for a hundred years. On the basis of not damaging local landscape resources, this study designed a conceptual effect map, as shown in Figure 7.

**Figure 8:**
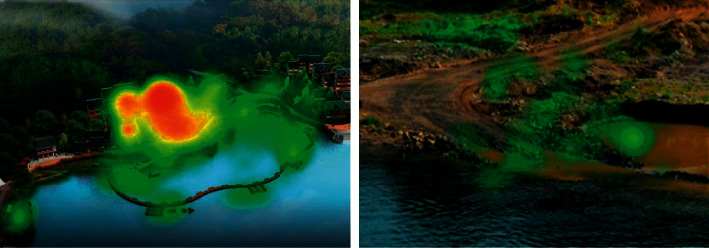
Exhibition of water culture tourist destination before and after reconstruction.

Mountain landscape attraction is mainly manifested in the following three aspects, Firstly, the mountain landscape environment is different from Binhai lakeside hot spring tourism real estate of another type, such as relying on the unique mountain natural ecological environment to attract tourists. Secondly, mountain villa holidays, especially in scorching summer, became the most suitable resort mountain. Thirdly, culture and entertainment are the soul of mountain tourism real estate. In order to make the development of mountain tourism real estate obtain lasting attraction, there must be a certain cultural environment, such as folk customs, temples, and historical sites. Entertainment mainly refers to the supporting facilities of tourism sites, such as commercial streets, hotels, and ski mountain bike races, which make tourism real estate sufficiently attractive and serve tourists better and drive the development of local economy. Exhibition of water culture tourist destination before (b) and after (a) reconstruction is given in Figure 8.

## 5. Conclusions

Through consulting a large number of books and literature, in the comprehensive use of landscape ecology, tourism, geography, biology, and other multidisciplinary theoretical knowledge, based on the research of 3D image technology, the study explores the related technologies and theories of combining mountain water cultural tourism real estate development with landscape planning and design and makes relevant explorations and studies on the problems existing in related concepts, content, technology, environmental conditions, and other aspects of planning. The research of this study will expand the research scope of mountain culture tourism and has great theoretical value and potential application prospect.

## Data Availability

The dataset can be obtained from the corresponding author upon request.
